# In this month’s Bulletin

**DOI:** 10.2471/BLT.18.000518

**Published:** 2018-05-01

In editorials, Monica Di Luca et al. (298) argue for earlier diagnosis and treatment of disorders of the brain. Duane J Gubler (299) describes the implications of further spread of yellow fever.

Sam Loewenberg (302–303) reports on the challenges faced in Zambia’s attempt to eliminate malaria. Yogan Pillay talks to Fiona Fleck (304–305) about South Africa’s moves to integrate service provision for tuberculosis and HIV.

## Argentina, Belarus, Brazil, Canada, China, Czechia, Costa Rica, Dominican Republic, Guatemala, Guyana, Ireland, Mexico, Mozambique, Nicaragua, Panama, Republic of Korea, South Africa, Sweden, Switzerland,Trinidad and Tobago, United Republic of Tanzania

### Just one drink 

Cheryl J Cherpitel et al. (335–342) test the validity of coding for a single episode of alcohol use resulting in injury.

## China

### Pesticides, narcotics, alcohol and psychodysleptics 

Lijun Wang et al. (314–326) track deaths from poisoning by intent and substance from 2006 to 2016.

## Japan

### Advocating for universal health coverage

Haruka Sakamoto et al. (355–359) describe efforts to influence policy at meetings between governments of Canada, France, Germany, Italy, Japan, the United Kingdom of Great Britain and Northern Ireland and the United States of America.

## Mexico

### Counting cases in an unprecedented outbreak

Juan Eugenio Hernández-Ávila et al. (306–313) estimate Zika virus infections.

## South Africa

### Zero deaths from rabies

K LeRoux et al. (360–365) report lessons learnt in a rabies control programme.

## Global

### Crowdsourcing disease surveillance 

Taryn Silver Lorthe et al. (327–334) evaluate an outbreak verification system.

### *Aedes* and aircraft

Shannon E Brent et al. (343–354) examine the potential for urban introduction of yellow fever virus by international travellers.

### 150 diagnoses 

Andrew M Briggs et al. (366–368) call for action to reduce the global burden of musculoskeletal conditions. 

